# Predict2Protect: Machine Learning Web Application in Early Detection of Heart Disease

**DOI:** 10.7759/cureus.49305

**Published:** 2023-11-23

**Authors:** Ankita Mandal, Soma Pradhan

**Affiliations:** 1 Center for Medical Sciences, Mills E. Godwin High School, Richmond, USA; 2 Obstetrics and Gynecology, Bon Secours St. Mary's Hospital, Richmond, USA

**Keywords:** applications of ai, accessible medical simulation, risk assessment tools, heart disease, ai and machine learning

## Abstract

Across the world, there are few universal scenarios, but the pain of losing a loved one to heart disease is an exception and a reality shared by millions every year. Heart disease is the greatest killer in society today, and one prevalent root of this issue is untimely diagnosis, often caused by unsustainable costs and lack of accessible healthcare for underserved populations. Recognizing these disparities, the goal of this project was to create an easily available application and interface for all that accurately indicates one’s risk of heart disease.

To address this, a machine learning model, Predict2Protect, was built in Python. An open-source dataset compiled of 1025 patients of diverse backgrounds was scaled, adjusted to include inquiries answerable by patients, and split into 75% for training, 15% for validation, and 25% for testing. Four models were tested with the hypothesis that if the RandomForestClassifier was used, it would have the highest validity. This was not supported, as the DecisionTree model had a 100% accuracy for training data and 95% for test data.

Through the application software Streamlit, this program was processed into a web application that is now found in browser extensions. The application reports the risk of one having heart disease with a 95% accuracy and describes the risk percentage of developing heart disease within the next year. With a simple interface and high accuracy, Predict2Protect aims to provide a view into one’s health with the goals of accessible heart disease prediction and early treatment for patients around the world.

## Introduction

Every 34 seconds, another individual’s life is claimed by heart disease, a condition characterized by threatening numbers and one that has increasingly become a killer at large. As the leading cause of death throughout the world, heart disease has claimed innumerable lives and continues to at this very moment, a reality faced by families around the world that have been plagued further by acute shortcomings in the form of accessible healthcare resources [1]. The availability and widespread distribution of healthcare in third-world and developing countries are scarce, and the threat of unreliable medical care is only exacerbated by issues of poverty and financial instability. Even within developed countries, healthcare is often infringed upon by the burdens of inadequate scheduling and unaffordable costs for the average citizen [2].

Without proper screening and regular medical visits, early detection, and treatment of heart disease, one of the prime methods of diminishing the risks the condition carries, a number of people who succumb to heart disease are at a much higher rate than what they could be with modern technology. This led to the main question of this study: how can one use machine learning in an application to further access to heart disease prediction for patients? Recognizing the potential in providing accessible care for everyone, this project was created with the aim of predicting heart disease using the same indicators identified by healthcare professionals [3]. In this way, those with a family history of heart disease, pre-existing conditions, a lack of proper access to medical care, and even just a yearning to assess their health will have the ability to do so, a measure with the potential of saving one’s life. 

By utilizing existing datasets containing thousands of patients’ data from diverse backgrounds, four different data-fit models were used to create a machine learning program for heart disease prediction and protection. Therefore, the hypothesis was as follows: if four fit models are used to create a machine learning framework for heart disease prediction, then RandomForestClassifier will be the most accurate and successful. This was believed because of RandomForestClassifier’s ability to dissect larger datasets with greater accuracy in similar projects where trial points of up to three thousand participants were utilized [4]. Of all the data-fit models tested in these other projects, which included KNN and SVM that are to be tested in this project as well, RandomForestClassifier was able to take into account the most significant variabilities of the data and respond with the greatest accuracy [5].

A simple input of personal data from a patient is incredible in its capacity to help one determine their medical standing in terms of heart disease and decide what steps they would like to take since many manifestations of early heart disease are detectable through symptom description [6]. When given a platform to voice these symptoms, detection becomes much simpler. As such, the variables to be considered in this platform encompass the extent of the issue by considering the independent variable as the fit model used to frame the program. Specifically, KNN, SVM, DecisionTree, and RandomForestClassifier were tested, as they were found to be the most accurate and well-fitting models for data with specific characteristics and varied scales as applied in the utilized dataset [7]. Each model has its own rate of success and includes varying degrees of fit. Based on these measures, the program will interpret user input in varying manners and create models of different accuracies. 

It is also vital to consider the true meaning of each of the diagnostic results from testing different fit models since, even if they are high, they may not be accurate in representing the true nature of the model. To prevent such issues, the data will be split into test and train sets with an extra validation set for confirmation of the initial train data [8]. The result of the independent variable changing is a greater overall difference in the accuracy of the model, and this is considered the dependent variable. As the accuracy of the model fluctuates in the presence of various models, the program can be evaluated for its applicability. 

Control in this project could be considered as the results of each model’s validation set of data as this set the basis of comparison between the original train data and testing data, but there was not a true control group because no model could be used against all others. However, to counteract this deficiency, 1025 repeated trials were conducted for maximum accuracy and allowance for the greatest fit of the data, and the factors considered, number of repeated trials, scaling of data, and amount of data separated into test, train, and validation were all kept constant to maintain consistency in the models [9]. These data points were collected with diversity in race, gender, and age, which are represented as user input options that will allow the model to take into account historical trends for varying backgrounds. These data will be kept confidential and can be left blank with a notification that this may change the accuracy of their results. 

As a web application, this software will be accessible and recommend medical help to patients, if necessary, within healthcare facilities. However, prior to the publication of this application, it will need to be tested in a monitored clinical setting around medical professionals for validity. This will be done to prevent the overuse of medical resources and avoid generating excess public concern. For this reason, Predict2Protect has been instated in a medical setting for six months and will continue to be for the next 2.5 years in order to collect patients and assess its validity with healthcare professionals present. This will allow for maximum accuracy for data collection itself and the appropriate use of medical resources. Heart disease has taken the lives of many, but as technology evolves, humans’ response to it must as well, and this project takes a stride in that direction.

## Materials and methods

First, a dataset had to be acquired. Using the platform Kaggle, a dataset was found from user David Lapp, and this dataset was selected with certain characteristics that indicated reliability. Firstly, the data were compiled from four widely varying areas: Cleveland, Hungary, Switzerland, and Long Beach. This allowed for variation in the data since concentrating results on a certain group would limit the data. This dataset had over 60,000 downloads and was published just four years ago, indicating a high efficacy rate with recent data [10]. Libraries such as NumPy, Matplotlib, Pandas, and Scikit-learn were imported into the Jupyter Notebook as the program was written in Python.

**Figure 1:**
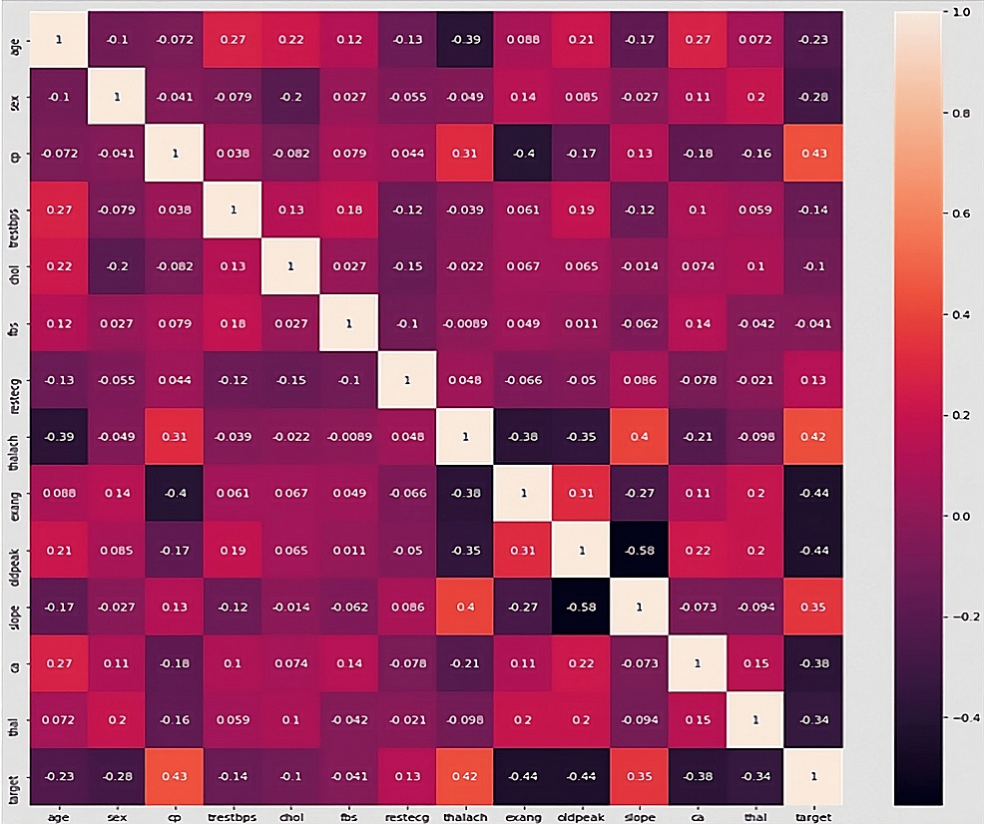
Correlation matrix for relationships between all factors

The dataset of 1025 patients of various ages, genders, and backgrounds was then separated into training data and testing data, from which the information was fed into a machine learning model that incorporated 13 factors referenced in the image above could be measured, four of which can be found using an ECG and were therefore excluded from the final product due to lack of access to proper equipment in the majority of areas. The remaining factors are age, sex, chest pain type, resting blood pressure, cholesterol levels, blood sugar, resting heart rate pain levels, maximum heart rate pain levels, and exercise-induced pain levels. The pain levels were all standardized to a scale of 10 based on possible responses, and this was reflected in the overall scaling process. For example, moderate heart pain in response to exercise could be selected in a dropdown menu, and in the program, this was interpreted as 5 on a scale of 1-10. With the use of exploratory data analysis (EDA), the data were separated, and the model was run. The correlation matrix shown in Figure 1 was created to assess the validity of each factor, and it was found that each attribute contributed to the validity since matrix scores varied significantly.

**Figure 2:**
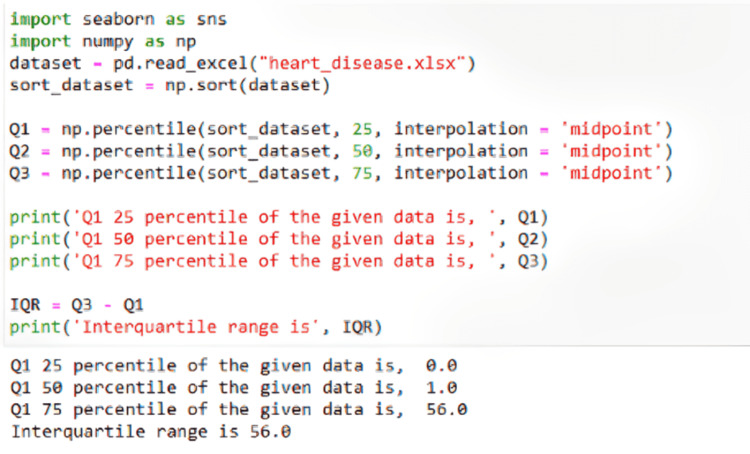
Standard scaling code for factor 4

Then, the data were scaled with the code shown in Figure 2 to allow the model to understand the significance of different values, such as when Boolean values were entered versus standard integer inputs. To determine whether it was as sufficient and indicative as possible, the data were split into X and Y sets first, where X contained all the attributes tested upon to find Y, the data containing whether or not one had heart disease. From here, the data were further split into 75% training and 25% testing data to ensure that the model would have new inputs to test once the model had been trained. To ensure that the training data were also adequately tested, this X set was also split to contain a validation set with which the model could be checked once more after the initial training.

The models were then run on the training, validation, and testing set chronologically and analyzed for the highest accuracy rate. Refining of each model was performed accordingly, such as post-pruning for the DecisionTree model. Then, the model to be used in the program was decided to be the DecisionTree model due to its high accuracy rate from classification reports run within the program. A t-test statistical analysis was performed in support, taking into account the accuracy averages of all the models, and proving the statistical significance of DecisionTree.

Following this, an extra measure was made to transform this model from a program to a widespread application. The location for this program was chosen to be a mobile application that can be reached in diverse areas, as is the purpose of this study.

Using a pickle file, the program containing the machine learning model was then imported to Spyder for processing into a mobile application. The interface of the application was designed with the software Streamlit, which was also imported into Spyder. From here, a series of inquiries requesting user input were formulated based on each attribute from the dataset. An additional probability function was added to the program in order to process the development of heart disease within a year of input. This was based on the use of the time progression model from StatsModels library that used the correlation of age, symptoms, and positive heart disease outcomes, and by considering one’s age one year after their inputted age combined with symptoms, the probability for their risk for positive heart disease was indicated with a percentage. Personal data, such as name and email, or further details to easily identify a user were not inquired in this program to maintain maximum privacy.

## Results

When each of the decision techniques for the data was run, there was a slight difference between the average accuracy rate of the model between training and testing data, and this is to be expected as the data fluctuate and increase the expanse of the model’s capabilities. However, there was a clear forerunner despite this variation. Referencing Table 1, the DecisionTree model was observed to have the highest validity of all the models, with a 100% accuracy with training and validation datasets, and when the testing set was factored in, the accuracy dipped ever so slightly to 95%. SVM, KNN, and RandomForestClassifier had accuracy rates of 69%, 84%, and 77%, respectively, with only the training data considered, which clearly revealed the DecisionTree model to be the most reliable.

**Table 1:** Classification report of DecisionTree model for training and validation dataset

Classification report categories	Precision	Recall	F1-score
Training data	1	1	1
Validation data	1	1	1
Accuracy			1
Macro average	1	1	1
Weighted average		1	1

From this analysis, the DecisionTree model was able to determine the requirements to qualify for a person who was at risk for heart disease and differentiate these results from one who was not at the same risk. There is an issue of the over-fit present with the validation data factored into the results of Table 1, but this was counteracted by the consideration of testing data that reduced the model’s accuracy to the aforementioned 95%, a probability level that is based on thousands of trials and relays a high degree of reliability. However, the work could not stop here, as this model was meant for more accessible use than an encoded program, and with this considered, the process of converting this program into a usable mobile application ensued. As for the other models, there were consistent discrepancies in the classification reports conducted for data analysis that lowered accuracy. 

As identified in the classification report of Table 2, the RandomForestClassifier model, which is recognized for its ability to analyze and conform to data, was not nearly as accurate as the DecisionTree model. The accuracy rate of 77% indicates that the model struggled to understand how often to predict heart disease based on the given systems and how correct these predictions were. The model’s confusion was also present in the KNN and SVM models, which were both also weighed down by a lack of precision that, in turn, contributed to a lack of success in identifying heart disease. Verifying that these results were consistent with the data was made possible through a t-test. 

**Table 2:** Classification report of RandomForestClassifier for training and validation data

Classification report categories	Precision	Recall	F1-score
Training data	0.79	0.84	0.83
Validation data	0.74	0.62	0.76
Accuracy			0.77
Macro average	0.75	0.78	0.80
Weighted average	0.77	0.77	0.81

Using Python and the imported libraries, a t-test was run between the average accuracies of the data using NumPy, pandas, and SciPy Stats for an alpha of 0.05. The null hypothesis for this condition was as follows: there is no significant difference between the averages of accuracy for each fit model. Inputting the average accuracies and applying the t-test function, the test statistic was found to be −133.334, and the p-value was calculated to be 0.0035, therefore rejecting the null hypothesis and indicating a high statistical significance for the data. This supports the conclusion that the DecisionTree model is highly effective and suitable for analyzing patient input in conjunction with heart disease detection and predictions.

## Discussion

Understanding the results of this project is critical to successfully implementing this application. The final conclusion to be drawn from testing the different fit models is that the DecisionTree model is the evidently best-fitting model for Predict2Protect’s program with an accuracy rate of 95%. Therefore, the hypothesis stating that RandomForestClassifier would be the best fit is not supported. Despite the high accuracy of the DecisionTree model, there are several factors taken into account to caution against the historical principles of the model itself, and keeping these factors in consideration is what creates the strength of this application’s program [11]. 

A study recently established a heart-disease prediction program with an extremely similar dataset but instead employed SVM, Gaussian Naive Bayes, logistic regression, and RandomForestClassifier [12]. Here, the accuracy rates produced from running each model over the refined dataset were 80.32%, 78.68%, 80.32%, and 88.5%, respectively. In this scenario, RandomForestClassifier was found to be the highest ranking in terms of accuracy and represented an overfit of 100% at first when run on only the training data, which is identical to how DecisionTree reacted in this project [13]. However, the difference lies in DecisionTree’s maintenance of the accuracy rate. Although RandomForestClassifier remains highest for the dataset used in this particular study, DecisionTree offers a more accurate view of the same prediction goal. The difference in RandomForestClassifier’s accuracy rate between studies can be attributed to disparities in the datasets used. 

There was a nuance addressed earlier in the making of this application that allowed for further accessibility regardless of resources; several of the inputs requested were to be gathered from a recent electrocardiograph (ECG) screening, and this was included in the original dataset for those who may have had this screening done but did not approach further medical aid or were not able to due to reasons of finance, accessibility, etc. However, this project is aimed particularly toward groups with populations who may not have available results from an ECG, as these tests can range from $150 to $300, and considering this, these questions were removed [14]. The program maintained its accuracy rate for the DecisionTree model, but this could be a potential source of error and difficulty for the other models as they may have functioned better with respect to more specific ECG data.

However, this did reveal the limitations of this study as well. To address the possibility of inappropriate overuse of this program, which would cause a potential drain on medical resources, this program required a clinical setting for preliminary use for the next two and a half years. This allows the program to accumulate the most correct data in the presence of medical professionals who have been able to identify the program’s accuracy. With this in mind, the program will gain patient data points and can be used independently on this track.

While more physical attributes such as age and gender could be easily found, it became difficult to incorporate more specific social factors that have proven increasing significance in heart disease development. Such factors include stress levels and daily responsibilities. This study was also limited by the aforementioned lack of ECG and medical screening equipment. This was lessened by adding more factors that sharpened the program, but improvements can also be made to the procedure of this project by further eliminating the risks of overfitting and even underfitting by dividing the data more to train the model continuously [15].

## Conclusions

Predict2Protect’s model is successful with 97% accuracy because the program is trained to detect symptoms of heart disease using indicators that can be found at home. The selected model, DecisionTree, is recognized for overfitting, as it can often become hyper-specific to the training data. In anticipation, the data were split into training, validation, and testing data to allow multiple opportunities to relearn the data. Post-pruning, or removing branches that became too particular to a narrow dataset, was also utilized to reduce overfitting. The program’s maintenance of its accuracy rate, even with these measures, displays strength and accuracy in prediction power. 

On the interface of this application, the user is informed of the likelihood of having heart disease within less than 10 seconds, and this output is framed with an additional probability function. This function assesses the possibility of developing heart disease within the next year, and this is a step toward prediction that allows users to plan. The patient is then informed to reach out to local medical care if concerns arise from the results. This is an aspect of the program that can be expanded in future research; for example, greater projections for the future, such as extending this time of projection for the probability of developing heart disease in the next five years. As this application gains use in its preliminary clinical setting, more data points are found, and the program gains accuracy in the presence of trained professionals. It is projected to have enough patient data for independent use in the next three years. This program is a step toward providing this personal assessment of one’s health to people across the globe. It is accessible technology like Predict2Protect that has provided the means to change lives forever.
